# Children's Motives for Admitting to Prosocial Behavior

**DOI:** 10.3389/fpsyg.2016.00220

**Published:** 2016-02-18

**Authors:** Yayoi Watanabe, Kayo Lee

**Affiliations:** ^1^Department of Psychology, Hosei UniversityTokyo, Japan; ^2^Graduate School of Humanities, Hosei UniversityTokyo, Japan

**Keywords:** development of prosocial behavior, admitting to prosocial behavior, children, motivation, honesty, lying, gender

## Abstract

There has been extensive research on children's moral evaluation of lying in prosocial situations. Current knowledge regarding the concept of lying has been derived from studies showing that cultural differences exist, whereby non-Western children tend to rate lie telling more positively than Western children do. These findings suggest that there are different views about whether children should publicize their prosocial behaviors and that children have universal motives when they admit to engaging in prosocial behavior. A gender difference has also been found in relation to prosocial behavior. However, previous studies did not investigate in detail children's motives for admission or non-admission to prosocial behavior, and if there is a gender difference. Therefore, this study examined the diversity in and development of motives for admitting or not admitting to engaging in prosocial behavior, with the aim of clarifying these behaviors as a function of children's grade level in school, and how such motives differ with age and gender. Questionnaires from 1345 elementary and junior high school students in Japan were analyzed. Results showed that children's communication tendency with regard to prosocial behavior reports peaked in the fourth grade of elementary school and gradually decreased thereafter. From the third grade of elementary school onwards, children reported that they refrained from admitting prosocial behaviors. Younger children more likely cited honesty as a crucial motive for admitting to prosocial behaviors. Girls were more likely to endorse honesty as a motive than boys were. Moreover, among younger children, girls feared others' negative evaluation and wanted to comply with modesty norms when not admitting. Further research is needed to examine the developmental process for motives behind prosocial behaviors.

## Introduction

In elementary and junior high schools throughout Japan, *telling the truth* and *refraining from telling lies* are considered essential moral values for children (Ministry of Education, Culture, Sports, Science, and Technology—Japan, 2011a,b). Likewise, in home-based education, refraining from telling lies is considered an important moral value to instill in children (Murayama, 1988).

With regard to research on morality, Piaget's (1932) work on lying and truth telling is widely known. Piaget presented children with stories involving lying as well as clumsiness and stealing in order to examine the relative importance that children place on motives and consequences. However, rather than focusing on the act of lying itself or the context of such an act, Piaget only considered lying as one of the values comprising a person's overall moral consciousness, that is, his or her sense of right and wrong.

This initial research on truth telling was built upon a new wave of studies conducted in the 1990s (see e.g., Sodian, 1991; Peskin, 1992; Russell et al., 1994). Notably, these researchers did not study lying in relation to judging right from wrong but instead examined two main trends, comprising the use of lying to deceive others and lying with a prosocial behavior motive, that is, to help others. Studies of the former trend focused on what skills are necessary to deceive others, that is, to lie successfully and the cognitive development of such skills. Studies that took the latter approach examined lying (or omission of truth) in a situational context, where the protagonist spontaneously performed a prosocial act (such as wiping down a dirty table) but did not admit to doing this when someone later asked who did it. These studies were focused on exploring the motive behind such social behavior.

This study takes its cue from the second research focus to answer the question of why an individual who spontaneously committed a prosocial act to help others would deliberately avoid admitting to such an act. There are two main explanations regarding the motive for not admitting to prosocial behavior. The first is *compliance with social norms* (Lee et al., 1997; Heyman et al., 2011) and the second is *impression management* (Yoshida et al., 1982). Lee et al. (1997) targeted compliance with social norms in a sample of 7-, 9-, and 11-year-old Canadian and Chinese children by presenting stories in which the protagonist does not admit the truth about having performed an antisocial or a prosocial deed. The children rated the protagonist's behavior on a seven-point scale ranging from “very, very bad” to “very, very good,” and the results revealed a cross-cultural difference in answers between the Canadian and Chinese children in the case of prosocial lie telling only. The Chinese children positively rated the characters who did not admit to having performed a prosocial deed and were more likely to deny having performed such a deed themselves as they got older. On the other hand, the Canadian children tended to give more negative ratings of the lie-telling protagonist. Moreover, Lee et al. (2001) presented similar scenarios involving lying in a prosocial context to Taiwanese and Japanese children, and their results corroborated the earlier findings that Asian (Taiwanese and Japanese) children were more likely than Canadian children were to positively rate denials of prosocial behavior, and that this tendency increased with age. Lee et al. called this tendency to deny prosocial behavior the *modesty effect* and explained it as the internalization of the social norm of modesty, which is an attitude often observed in interpersonal settings in Eastern cultural contexts.

However, it is unclear from this previous research as to whether or not the children actually had such a mindset, as they were never directly asked about their motives for not admitting the deed to others.

Other scholars have suggested that individuals avoid admitting to prosocial behavior not for reasons of modesty, such as not wanting to boast about one's good qualities or performance, but rather as an impression management strategy, whereby they voluntarily avoid telling others about their good points with the expectation of receiving a positive evaluation from others (Aikawa, 2003; Yamaguchi and Tafarodi, 2004). According to Yoshida et al. (1982), by the second year of elementary school, children have already started favorably assessing the character traits of individuals who express their character in a reserved manner and have reached an understanding of impression management.

Thus, this study takes the view that it is reasonable to postulate concern about others' evaluations and consideration about others as alternative possible motives to those suggested in the past research (i.e., compliance to social norms and impression management). The existence of these two motives has also been noted in a number of preceding studies. For example, Banerjee (2000) conducted a study on concern about others' evaluations by presenting two types of scenarios to 6- to 10-year-old children that involved protagonists receiving praise for achieving a good result. In the first type, the protagonist was praised by the teacher for getting an excellent result in a mathematics test, while in the second type, the protagonist was praised by the teacher for excelling at catching a ball during a physical exercise class. The study inquired into the responses the children would give to the teacher and the results revealed that the children were very concerned about not being thought of as boastful. It was also pointed out that this tendency to give unassuming responses became more pronounced with age. Murase (2000) showed that a characteristic of elementary school children is their observance of group norms and unwritten rules, suggesting that children will try their best to avoid actions that make them stand out from a group. Furthermore, research on developmental changes connected with the concern about others' evaluations indicated that this type of concern increases at the junior high school stage irrespective of gender (Yamamoto and Tagami, 2007). This finding suggests that during this period of powerful group conformity tendencies, children are loath to do anything to stand out or make others envious.

As for the motive of concern for others, Han (2010) described this as the assumption that being singled out for praise would indirectly indicate that the surrounding people are not being praised. Han predicted that the individuals concerned would avoid such a situation for fear of hurting the feelings of others. For example, suppose Child A gets praised by the teacher for cleaning the classroom. Such praise would indicate that Child B and Child C did not clean the classroom, making it possible that Child B and Child C would be uncomfortable and get upset. It is, therefore, assumed that Child A, in not wishing to stand out, would avoid admitting to having done the cleaning. Such a capacity is thought to be related to *social awareness* and *empathy* (Eisenberg et al., 2006). According to Selman (1981, 2003), children around the third year of elementary school have reached the second stage of role taking, *self-reflective role-taking*, which means that they can distinguish the perspectives of others from their own, put themselves in the position of others, and reflect on their own thoughts and feelings accordingly. Selman (2003) argued that the ability to understand and coordinate one's own perspective with that of another person is a fundamental determinant of moral and social development in children. The skills of taking perceptual, affective, and cognitive perspective have been shown to be positively related to prosocial behavior. As pointed out by Han (2010), children from the third year of elementary school onward acquire the tendency to show concern for the feelings of others and to avoid accepting praise unreservedly. Therefore, it seems reasonable to posit the existence of concern for others when conducting such research with elementary school-age children.

However, research thus far on prosocial behavior has suggested a variety of motives behind lying or not telling the truth, but few studies have considered the full range of options or directly inquired into the motives. Moreover, the developmental changes that come with age have not yet been studied. To address this shortcoming in both types of study, Lee (2011) surveyed 1st, 3rd, and 5th year elementary school students, presenting the children with the same types of scenario used in Lee et al. (1997, 2001) and asking them to give free, descriptive answers about their reasons for not admitting to performing a prosocial deed. Students were directly asked about the motives behind their prosocial acts. Lee (2011) found that there were nine categories behind admitting to prosocial acts and nine other categories behind not admitting to prosocial acts. Younger children just asserted their judgments as their motives. Some children feared their friends' malicious feelings, and other children responded with altruistic motives. As children grew up, they could better clarify their motives by various expressions and describe more precisely how they considered others' points of view. The study found that there were new motives that had different nuances and detail information in their expressions that looked similar. The study did not distinguish between children's expressive skills and their motives.

Therefore, this study examined the detailed motives for not admitting and for admitting to having performed a prosocial act in addition to the motives, such as concern about others' evaluations, concern for others, compliance with social norms, and impression management, which were suggested in past studies. This study chose the typical eight categories of motives (four for admission and four for non-admission to having performed a prosocial act), because all children can understand and choose these motives despite their lacking competence to express their motives. The choices revealing underlying motivations were selected from children's responses in Lee (2011).

Considering the above, it seems to be important to consider “concern about others” as a motive for not admitting to engaging in prosocial behavior, and to conduct an examination of children who are older than those targeted in the study by Banerjee (2000). Importantly, the aims of this study did not include examining the relations between children's motives and moral values, because Lee (2011) had admitted that distinguishing between motives and values was difficult. Furthermore, while scenarios used in previous studies only involved either non-admission or admission of the protagonist, this study also inquired into the basis for the decision and focused on cases where individuals frankly admitted to performing prosocial behavior as well as cases where they did not.

The current study focused on the motives behind admission or non-admission decisions. Moreover, this study aimed to clarify age-related developmental changes in terms of the decision to admit or not admit to prosocial behavior and the motives behind such a decision. Since the age of the studied children varied across the aforementioned studies, it was unclear whether there are developmental changes. In addition, this study is an extension of previous research by investigating whether there is a gender difference in making these admissions. Our results should shed light on the development and diversity of children's motives regarding admission and non-admission of performing prosocial acts.

## Material and methods

### Participants

A questionnaire survey was conducted with a sample of children from five public elementary schools and one junior high school in the Kanto region of Japan. Participants were 1345 children in academic years (grade levels) ranging from 3rd grade of elementary school to 2nd grade of junior high school. The analysis was conducted on 1212 of these children, after removing from the analysis surveys containing incomplete or defective answers. The breakdown by grade was as follows:

Elementary school 3rd grade (between 8 and 9 years): 246 students (130 male, 116 female)
Elementary school 4th grade (between 9 and 10 years): 233 students (119 male, 114 female)
Elementary school 5th grade (between 10 and 11 years): 195 students (102 male, 93 female)
Elementary school 6th grade (between 11 and 12 years): 274 students (144 male, 130 female)
Junior high school 1st grade (between 12 and 13 years): 128 students (70 male, 58 female)
Junior high school 2nd grade (between 13 and 14 years): 136 students (73 male, 63 female)

All study participants provided informed consent, and Hosei University's ethics review board approved the study, which was conducted in 2011.

### Stimuli

Two prosocial behavior scenarios were prepared. In contrast to preceding studies, which used scenarios involving a third-person protagonist, this study used scenarios in which the subject was not a specific person, but rather “you,” in order to make it easier for the participants to identify with the protagonist's point of view.

In order to eliminate terms that might have sounded unnatural, depending on the school the participants attended, the term “morning assembly” was used in questionnaires sent to the elementary schools, and “homeroom period” was used in questionnaires sent to the junior high schools.

#### Scenario one: wiping the blackboard

Your class at school has just finished. You notice that the teacher's writing on the blackboard hasn't been wiped off yet. It's not your turn to be in charge of blackboard duty, but you wipe the writing off the blackboard anyway and head home. One of your friends, who was in the classroom at the time, noticed you doing this. The next day, during morning assembly (homeroom period), your teacher says the following: “I heard that someone wiped the blackboard yesterday. Who did it?”

#### Scenario two: closing the window

Your class at school has just finished. Just as you're about to head home, a heavy rainstorm begins. It's raining hard, so to prevent the classroom from getting soaked, you close all the windows before heading home. One of your friends, who was in the classroom at the time, noticed you doing this. The next day, during homeroom period (morning assembly), your teacher says the following: “It rained hard after class yesterday, didn't it? I heard that someone closed the windows. Who did it?”

### Procedure

In the questionnaire, participants were asked to choose whether or not to admit to having performed the deed, and then to rate each of the possible motives for making such a decision. The questionnaire was self-completed by the students, and the specific details are as follows.

#### Selecting admission/non-admission

The participants read the scenario about “them” having performed a prosocial deed and being asked, “Who did it?” by the teacher, before being prompted to give a response. The specific question they were asked was as follows: “*Would you say, ‘I did it?”’* There were two possible answers, that is, “*Yes, I would say that”* and “*No, I wouldn't say that*.”

#### Rating motives for admission/non-admission

After they had chosen whether or not to admit to having performed the deed, the participants were presented with a number of items stating possible motives for admission and non-admission (see Table 1). The participants were prompted to rate these items on a five-point scale, where 1 was “I do not think this at all” and 5 was “I think exactly this.” It was anticipated that there might be other possible motives in addition to those stated in the items, and so the participants were asked at the final stage to provide a free descriptive answer. Furthermore, the order of these items was counterbalanced in order to reduce the effects of the order influencing the results. Moreover, participants could add their unique motivations into the open text fields if they did not find any of the motivations in the prepared items to be appropriate.

**Table 1:** **Content of items regarding possible motives for admitting or not admitting to performing the prosocial deed**.

**Motives for admission**	**Description of motive for admission**
Goodness of prosocial behavior	(a) I did a very good deed that is well-worth telling the teacher about.
Emphasis on honesty	(b) It's best to tell the teacher that I wiped the blackboard (closed the windows), because that's the honest truth.
Consideration of disturbance to others	(c) If I don't answer the teacher, my classmates will be left wondering who did it.
Pursuit of evaluation	(d) I did a good deed, and I want others to recognize that.
**Motives for non-admission**	**Description of motive for non-admission**
Impression management	(a) Others will think better of me if I don't tell.
Consideration of others	(b)-1 If I admit that I did it, then people will think my friend who was there at the time is careless.
	(b)-2 If I admit that I did it, then people will think the person who was responsible for this task didn't do their job.
Concern about others' evaluations	(c) If I admit that I did it, people will think I'm boastful.
Compliance with social norms	(d) The good deed I did was not great enough to deserve praise from my teacher and classmates.

*(b)-1 was used in scenario one, and (b)-2 was in scenario two*.

### Data analysis

Chi-square, Mann–Whitney, and Kruskal–Wallis tests were carried out. As these analyses involved a comparison among children across six grades, the Bonferroni correction was performed to adjust the *p* values obtained from the Mann–Whitney test. Multiple comparisons were then carried out.

## Results

### Admission/non- admission

**Figure 1:**
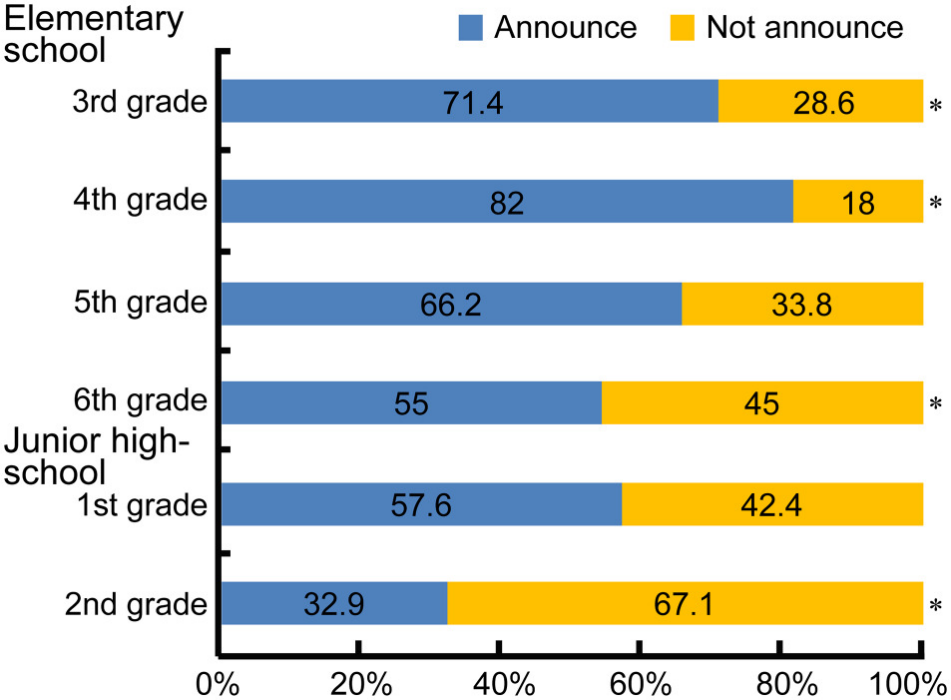
Ratio of grade levels of participants who selected “Admit”/“Not Admit” in relation to prosocial behavior. ^*^p < 0.01.

Chi-square (χ^2^) tests were used to analyze the decision of whether or not to admit to engaging in prosocial behavior. The results revealed a significant difference across a number of participants, χ${}_{\left(5\right)}^{2}=98.507$, *p* < 0.01. Residual analysis revealed that more elementary 3rd and 4th graders opted to admit to prosocial behavior compared to those in other grade levels (see Figure 1). However, after peaking at elementary 4th grade, the answer rate declined, and the ratio of those who opted not to admit rose in elementary 6th grade and junior high 2nd grade. No gender-based difference was observed, χ${}_{\left(1\right)}^{2}=0.707$, *ns*.

### Motives for admission of engaging in prosocial behavior

#### Differences in admission motives based on grade level and gender

**Figure 2:**
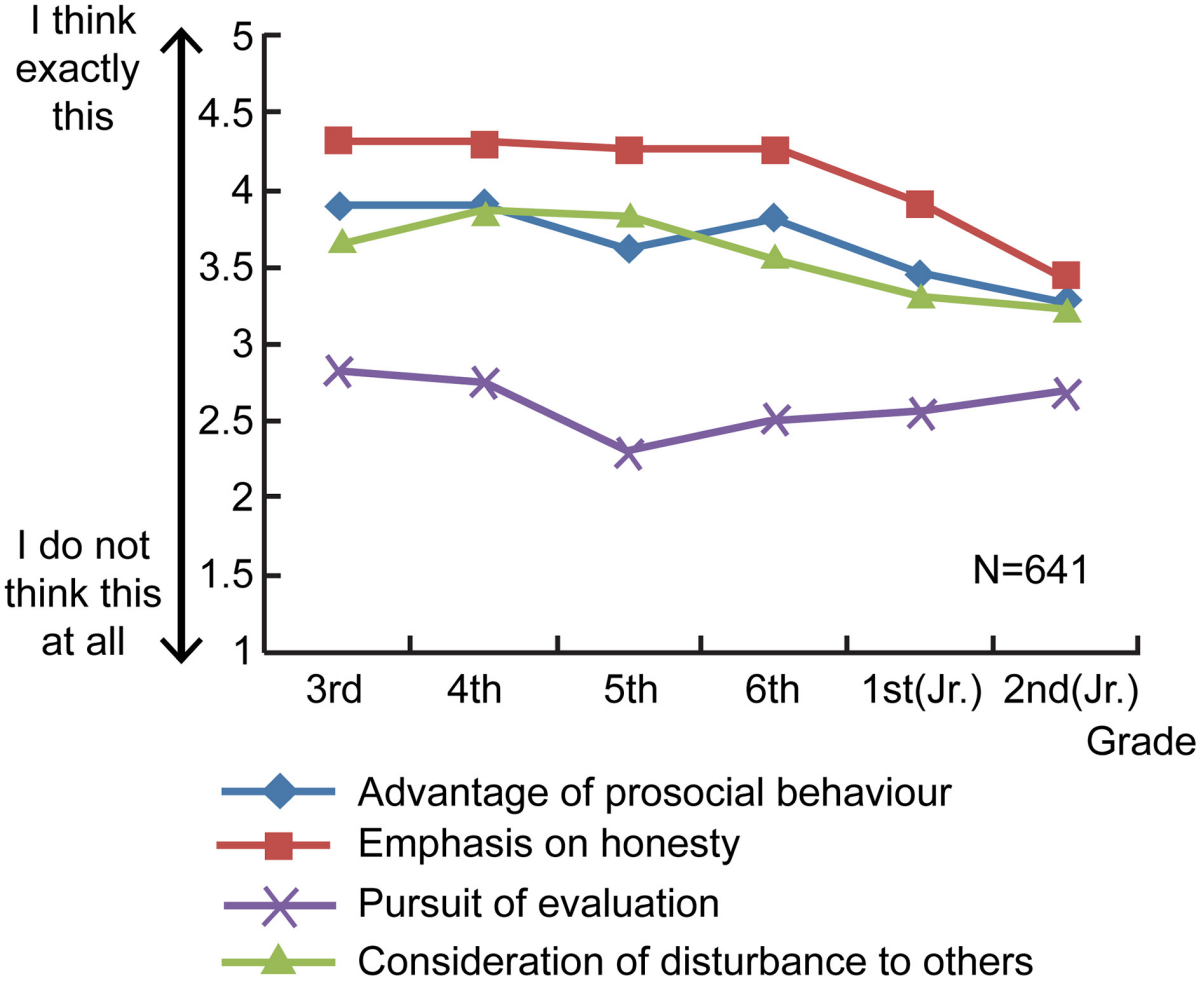
**Average admission ratings across each grade level and motive (motives for admission)**.

Figure 2 and Table 2 show the average admission ratings across grade level and motive. In consideration of the fact that we used an ordinal rating scale, a non-parametric Kruskal–Wallis (λ) test was performed on the four motives of admission. With the exception of pursuit of evaluation, there were significant differences across grade levels (advantage of prosocial behavior: χ^2^ = 20.17, *df* = 5, *p* < 0.05; emphasis on honesty: χ^2^ = 29.27, *df* = 5, *p* < 0.001; consideration of disturbance to others: χ^2^ = 15.64, *df* = 5, *p* < 0.05; pursuit of evaluation: χ^2^ = 5.13, *df* = 5, *ns*). As stated above, the Bonferroni correction was performed to adjust the *p* values obtained from the Mann–Whitney (*U*) test, before multiple comparisons were carried out. The results revealed that emphasis on honesty was the only motive for which scores varied according to grade level. Specifically, elementary 3rd graders were more likely to score emphasis on honesty highly, compared to junior high 1st and 2nd graders, with median values as follows: elementary 3rd grade: 134.75; junior high 1st grade: 101.93, *U* = 5265.50, *p* < 0.05; elementary 3rd grade: 119.21; junior high 2nd grade: 76.33, *U* = 2413.50, *p* < 0.01. On the other hand, junior high 2nd graders did not rate emphasis on faithfulness as highly as elementary 4th, 5th, and 6th graders did, with median values being as follows: junior high 2nd grade: 82.92; elementary 4th grade: *U* = 2696.50, *p* < 0.01; junior high 2nd grade: 65.79; elementary 5th grade: 92.6, *U* = 1925.50, *p* < 0.01; junior high 2nd grade: 72.22; elementary 6th grade: 105.13, *U* = 2215.00, *p* < 0.001. In other words, the lower the grade, the more value was placed on honesty, but this attitude changed upon reaching junior high school, with honesty no longer believed to be always a good thing.

**Table 2:** **Descriptive statistics of each category in each grade**.

	**1**	**2**	**3**	**4**	**5**		
**MOTIVES FOR ADMISSION**							
**3rd grade**	**I don't think like this at all**		**Neutral**		**I think exactly like this**	***n***	**Mean (*****SD*****)**
Goodness of prosocial behavior	11 (6%)	13 (7%)	35 (20%)	58 (32%)	62 (35%)	179	3.82 (1.17)
Emphasis on honesty	3 (2%)	10 (6%)	17 (10%)	40 (23%)	105 (60%)	175	4.34 (0.99)
Consideration of disturbance to others	19 (11%)	11 (6%)	26 (15%)	58 (33%)	63 (36%)	177	3.82 (1.39)
Pursuit of evaluation	47 (27%)	26 (15%)	45 (26%)	37 (21%)	20 (11%)	175	2.76 (1.36)
**4th grade**	**I don't think like this at all**		**Neutral**		**I think exactly like this**		
Goodness of prosocial behavior	10 (6%)	8 (5%)	37 (21%)	63 (36%)	55 (32%)	173	3.83 (1.10)
Emphasis on honesty	6 (3%)	5 (3%)	23 (13%)	50 (29%)	90 (52%)	174	4.22 (1.02)
Consideration of disturbance to others	19 (11%)	14 (8%)	28 (16%)	43 (24%)	72 (41%)	176	3.77 (1.35)
Pursuit of evaluation	45 (26%)	37 (21%)	41 (23%)	30 (17%)	23 (13%)	176	2.71 (1.36)
**5th grade**	**I don't think like this at all**		**Neutral**		**I think exactly like this**		
Goodness of prosocial behavior	11 (9%)	15 (12%)	31 (25%)	40 (32%)	28 (22%)	125	3.47 (1.22)
Emphasis on honesty	4 (3%)	5 (4%)	21 (17%)	30 (24%)	65 (52%)	125	4.18 (1.06)
Consideration of disturbance to others	12 (10%)	8 (6%)	21 (17%)	41 (33%)	43 (34%)	125	3.76 (1.26)
Pursuit of evaluation	42 (34%)	20 (16%)	38 (31%)	14 (11%)	10 (8%)	124	2.44 (1.28)
**6th grade**	**I don't think like this at all**		**Neutral**		**I think exactly like this**		
Goodness of prosocial behavior	7 (5%)	8 (5%)	38 (26%)	46 (31%)	49 (33%)	148	3.82 (1.10)
Emphasis on honesty	1 (1%)	8 (5%)	14 (9%)	57 (39%)	68 (46%)	148	4.24 (0.88)
Consideration of disturbance to others	22 (15%)	7 (5%)	30 (20%)	47 (32%)	42 (28%)	148	3.54 (1.35)
Pursuit of evaluation	37 (25%)	25 (17%)	55 (37%)	19 (13%)	12 (8%)	148	2.62 (1.22)
**1st junior grade**	**I don't think like this at all**		**Neutral**		**I think exactly like this**		
Goodness of prosocial behavior	4 (5%)	7 (9%)	22 (30%)	31 (42%)	10 (14%)	74	3.49 (1.02)
Emphasis on honesty	1 (1%)	3 (4%)	20 (27%)	25 (34%)	25 (34%)	74	3.95 (0.95)
Consideration of disturbance to others	11 (15%)	7 (10%)	17 (23%)	23 (32%)	15 (21%)	73	3.33 (1.32)
Pursuit of evaluation	20 (28%)	10 (14%)	26 (36%)	14 (19%)	2 (3%)	72	2.56 (1.17)
**2nd junior grade**	**I don't think like this at all**		**Neutral**		**I think exactly like this**		
Goodness of prosocial behavior	6 (13%)	5 (11%)	14 (30%)	11 (24%)	10 (22%)	46	3.36 (1.30)
Emphasis on honesty	5 (11%)	5 (11%)	14 (31%)	7 (16%)	14 (31%)	45	3.44 (1.34)
Consideration of disturbance to others	7 (15%)	6 (13%)	13 (28%)	10 (22%)	10 (22%)	46	3.22 (1.35)
Pursuit of evaluation	10 (22%)	10 (22%)	13 (29%)	8 (18%)	4 (9%)	45	2.69 (1.26)
**MOTIVES FOR NON-ADMISSION**							
**3rd grade**	**I don't think like this at all**		**Neutral**		**I think exactly like this**	***n***	**Mean (*****SD*****)**
Impression management	23 (34%)	11 (16%)	19 (28%)	6 (9%)	8 (12%)	67	2.48 (1.36)
Consideration for others	18 (27%)	10 (15%)	16 (24%)	7 (10%)	16 (24%)	67	2.90 (1.52)
Concern about others' evaluations	14 (20%)	3 (4%)	10 (14%)	13 (19%)	30 (43%)	70	3.60 (1.55)
Compliance with social norms	7 (10%)	9 (13%)	16 (23%)	14 (20%)	23 (33%)	69	3.54 (1.35)
**4th grade**	**I don't think like this at all**		**Neutral**		**I think exactly like this**		
Impression management	20 (36%)	8 (15%)	22 (40%)	3 (5%)	2 (4%)	55	2.25 (1.13)
Consideration for others	16 (29%)	4 (7%)	14 (25%)	11 (20%)	10 (18%)	55	2.91 (1.48)
Concern about others' evaluations	6 (11%)	3 (5%)	11 (20%)	10 (18%)	26 (46%)	56	3.84 (1.36)
Compliance with social norms	3 (5%)	4 (7%)	17 (31%)	17 (31%)	14 (25%)	55	3.64 (1.11)
**5th grade**	**I don't think like this at all**		**Neutral**		**I think exactly like this**		
Impression management	39 (57%)	6 (9%)	18 (26%)	3 (4%)	3 (4%)	69	1.91 (1.60)
Consideration for others	28 (42%)	7 (10%)	11 (16%)	12 (18%)	9 (13%)	67	2.51 (1.51)
Concern about others' evaluations	18 (26%)	4 (6%)	9 (13%)	17 (25%)	21 (30%)	69	3.28 (1.59)
Compliance with social norms	9 (13%)	3 (4%)	17 (25%)	27 (39%)	13 (19%)	69	3.46 (1.23)
**6th grade**	**I don't think like this at all**		**Neutral**		**I think exactly like this**		
Impression management	47 (38%)	21 (17%)	34 (27%)	15 (12%)	8 (6%)	125	2.33 (1.27)
Consideration for others	32 (26%)	18 (14%)	36 (29%)	21 (17%)	18 (14%)	125	2.80 (1.37)
Concern about others' evaluations	18 (14%)	4 (3%)	21 (17%)	36 (29%)	46 (37%)	125	3.70 (1.37)
Compliance with social norms	9 (7%)	8 (6%)	41 (32%)	30 (24%)	39 (31%)	127	3.65 (1.19)
**1st junior grade**	**I don't think like this at all**		**Neutral**		**I think exactly like this**		
Impression management	23 (43%)	7 (13%)	17 (31%)	6 (11%)	1 (2%)	54	2.17 (1.16)
Consideration for others	13 (24%)	3 (6%)	15 (28%)	20 (37%)	3 (6%)	54	2.94 (1.28)
Concern about others' evaluations	9 (17%)	2 (4%)	7 (13%)	21 (39%)	15 (28%)	54	3.57 (1.38)
Compliance with social norms	1 (2%)	2 (4%)	19 (35%)	14 (26%)	18 (33%)	54	3.83 (0.99)
**2nd junior grade**	**I don't think like this at all**		**Neutral**		**I think exactly like this**		
Impression management	24 (27%)	12 (13%)	35 (39%)	12 (13%)	7 (8%)	90	2.62 (1.23)
Consideration for others	17 (19%)	6 (7%)	39 (43%)	22 (24%)	6 (7%)	90	2.93 (1.16)
Concern about others' evaluations	19 (21%)	4 (4%)	31 (34%)	24 (27%)	12 (13%)	90	3.07 (1.30)
Compliance with social norms	12 (13%)	5 (6%)	33 (37%)	23 (26%)	17 (19%)	90	3.31 (1.23)

With regard to gender differences in motives for admitting to prosocial behavior, the non-parametric Mann–Whitney test performed on each of the four motives revealed a significant gender difference in the median scores for emphasis on honesty. The median scores for this motive were 357.56 among boys and 393.76 among girls (*U* = 62909.0, *p* < 0.05). This indicates that girls were more likely than boys were to consider it a good idea to tell the honest truth about a desirable behavior they engaged in.

#### Differences in non-admission motives based on grade level and gender

**Figure 3:**
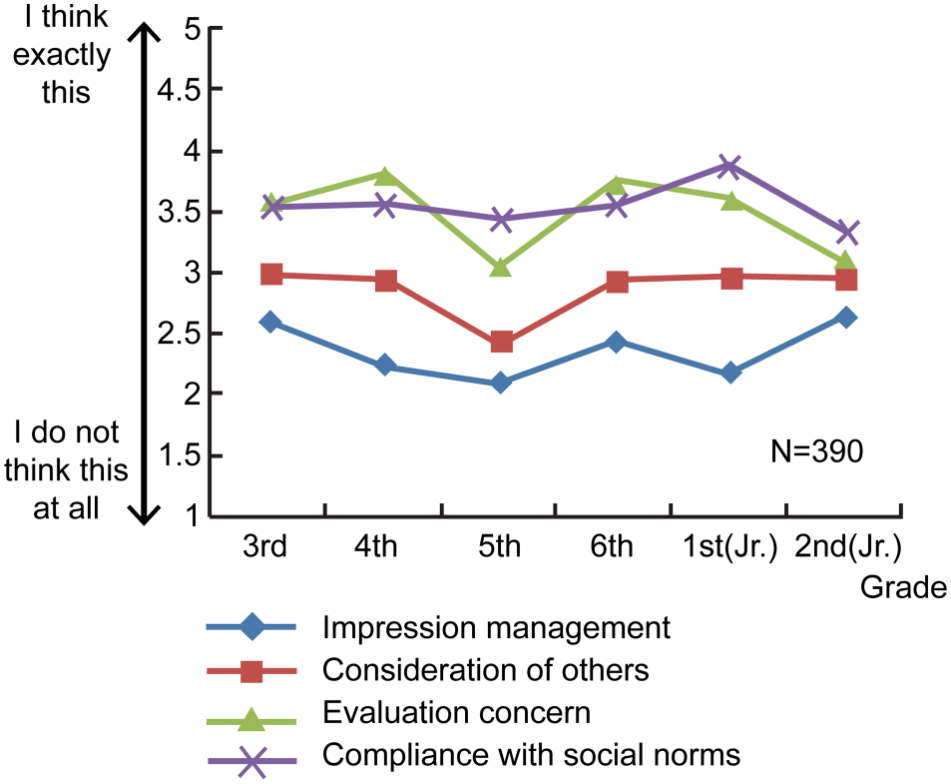
**Average admission ratings across each grade level and motive (motives for non-admission)**.

Figure 3 and Table 2 show the average non-admission scores across grade level and motive. The Kruskal–Wallis test was performed on the four motives for non-admission (i.e., impression management, consideration of others, concern about others' evaluations, and compliance with social norms). Significant differences were observed in the scores only for impression management (χ^2^ = 13.95, *df* = 5, *p* < 0.05) and concern about others' evaluations (χ^2^ = 20.51, *df* = 5, *p* < 0.01). The results revealed a grade-based difference in the scores for impression management only between elementary 5th grade (median value: 66.76) and junior high 2nd grade (median value: 91.19; *U* = 2188.0, *p* < 0.001). This shows that elementary 5th graders did not have a very positive view of using non-admission as a means of obtaining a favorable evaluation from others, and that junior high 2nd graders' view is less negative by comparison. There were significant differences in concern about others' evaluations between junior high 2nd graders (median: 63.86) and elementary 4th graders (media: 88.99, *U* = 1652.5, *p* < 0.001), and between junior high 2nd graders (median: 89.35) and elementary 6th graders (median: 121.43, *U* = 3946.5, *p* < 0.001). In other words, compared to junior high 2nd graders, many more elementary 4th and 6th graders chose non-admission out of concern of being negatively evaluated by others. With regard to gender differences in motives for not admitting to engaging in prosocial behavior, the Mann–Whitney test was performed on each of the four motives and did not reveal any significant gender-based differences. This finding indicates that boys and girls had largely the same motives for not owning up to performing a desirable deed.

## Discussion

The first aim of this study was to examine whether or not a person who performs a prosocial deed will admit to having done so. The second aim was to explore the motives behind the choice to admit or not admit to engaging in prosocial behavior. This study also aimed to investigate the developmental changes and gender-based differences in both cases.

From 3rd grade of elementary school to 2nd grade of junior high school, the tendency to admit to engaging in prosocial behavior reached its peak in elementary 4th grade and then declined with successive grade levels. In order to ascertain the reasoning behind the decision to admit or not admit to being prosocial, each motive was examined in terms of how it differed across grade level and gender. Although, this examination did not disclose any motives that became progressively popular with increasing grade level, it did reveal certain motives to be predominant among certain grades. For example, elementary students positively rated being honest about the deed they performed, while junior high students were less likely to give a positive rating. Furthermore, this tendency was more noticeable among girls than boys. As for the motives for not admitting to engaging in prosocial behavior, the results showed that junior high 2nd graders were less negative than 5th graders were about the idea of deliberately avoiding admitting to prosocial behavior in order to obtain a favorable evaluation from others (i.e., impression management). The results also revealed that elementary 4th and 6th graders were more likely than junior high 2nd graders were to be apprehensive about being negatively evaluated by others.

These findings corroborate those of Banerjee (2000), who found that when praised by the teacher, elementary school children, regardless of gender, were more likely to favor a self-effacing response than junior high children and children of lower grade levels were. In this study, we also found that junior high children were more likely to favor self-effacement as a means of impression management, which aligns with the results of Lee et al. (1997, 2001) and Lee (2011), who surveyed elementary children and found that children in higher grade levels were more likely to positively evaluate characters that denied having performed a good deed.

However, according to the results of this study, the year at which children gain a preference for not admitting to prosocial behavior is later than the year specified by Banerjee (2000), that is, the 3rd year onwards. The reason for this discrepancy probably lies in the type of scenario used. Specifically, the scenarios used in this study incorporated actions that take into consideration concern for others, and, compared to Banerjee (2000), there was a greater emphasis on the possibility of impacting one's surroundings (classmates, etc.) by admitting to prosocial behavior. According to Selman (1981, 2003), consciousness toward the self develops at an earlier stage than consciousness toward others and consciousness of self from another's perspective. This study presented situations that involved speculating on the feelings of others, which might explain why the year groups that favored non-admission were higher than in Banerjee's study. Moreover, Banerjee's participants were from the UK (i.e., a Western country), while participants in this study were from Japan (i.e., an Eastern country).

Yamagishi et al. (2012) showed that people from Asian cultures showed a self-effacing tendency, presenting themselves as less qualified and competent than others, compared with individuals from the US, which indicates that this may be attributed to cultural differences. To extrapolate the results of this study to daily life, it can be surmised that once they reach higher grade levels, elementary school children are much more likely to perform good deeds anonymously and cultural differences should be taken into consideration when examining these participants.

The only motive for admitting to prosocial behavior that varied according to grade level in this study was emphasis on honesty. Notably, junior high school children were much less likely than elementary children were to positively evaluate the notion that one should admit to prosocial behavior because it is right to tell the truth. A conceivable explanation for this finding is that with increased age-related development, children's means of admitting to prosocial behavior diversify. As pointed out by Maruyama (2009), who studied the development of self-assertive and self-inhibitory behavior, as children grow older, they gain the idea that they can convey their thoughts and feelings to others even without asserting themselves immediately. This study asked the participants to write down free, descriptive answers regarding possible motives that had not already been conceived of. Many different kinds of answers were given, including “The others will eventually realize what I did sooner or later, so there's no need to tell them right now.” These data suggest that as children age, they gain ideas and strategies for inhibiting admission behavior.

Children at higher grade levels were much more likely to avoid admitting to engaging in prosocial behavior, but as to the purpose for doing so, these children were more inclined to use non-admission as a means of obtaining approval or attention from others. The reason for use of this strategy at this stage could be that junior high school students have fewer opportunities to get attention by doing good things, and so they may have a greater desire to be thought well of. According to Kosaka (2008), junior high school students place great importance on such areas as academic results, which earn them praise from teachers or parents, because they perceive favorable academic results as an easy way to get praise. When junior high school students perform the kind of prosocial behavior described in this study's scenarios, there may be a strong tendency not to admit to the deed in the hope that they will thereby increase their chance of receiving praise.

However, with regard to the preceding research on impression management as a means of winning praise, most such studies (Lee et al., 1997, 2001; Banerjee, 2000; Lee, 2011) targeted young children. The results of this study revealed that a change in the motive for not admitting to engaging in prosocial behavior also takes place among junior high school students. In order to shed further light on age-related developmental changes, there is a need to broaden the focus to encompass high school students.

Another motive that was markedly preferred among the elementary children was concern about others' evaluations. As Murase (2000) mentioned, a characteristic of elementary school children is their observance of group norms and unwritten rules, and such children tend to be wary of the gazes of others and prefer to refrain from behavior that will make them stand out in a group situation. However, such a tendency does not become more pronounced as children develop; rather, it was found that the preference for concern about others' evaluations was weak among junior high children. This finding contradicts the results of Yamamoto and Tagami (2007), who found that concern about others' evaluations increases among boys and girls at the junior high school stage. This discrepancy might be due to the type of scenarios used, with this study using wiping the blackboard and closing the windows as examples of prosocial behavior. It is possible that the children regarded these examples as taking place in settings that had little bearing on their study life, and, as such, would have had a negligible impact on the evaluation they would receive from others. Therefore, the validity of these scenarios needs to be examined in the future.

In summary, this study revealed that children are more likely to refrain from admitting to prosocial behavior as they advance through the years, and that the motives behind such non-admission are diverse. It also revealed that children refrain from admitting to prosocial behavior in order to gain a good impression among others or to avoid negative evaluation, with these motives particularly noticeable among certain grades. After middle childhood, interpersonal problems such as bullying can arise. Conformity to deviant behaviors and social comparison in the classroom could be related to serious interpersonal conflicts. Moreover, intimacy exclusiveness and misconceptions about adolescent human relationships may influence attitude and social behaviors. However, this study was conducted in only one region in Japan; therefore, the findings should not be generalized to all schoolchildren in Japan. In terms of future issues, there is a need to further examine the appropriateness of admitting or not admitting to engaging in prosocial behavior, and how children can gain high moral standards. It is hoped that the current findings will contribute to a moral education or social emotional learning that gives consideration to children's levels of understanding.

## Author contributions

YW: Designed the work and drafted the manuscript. KL: Responsible for the acquisition, analysis, and interpretation of data.

### Conflict of interest statement

The authors declare that the research was conducted in the absence of any commercial or financial relationships that could be construed as a potential conflict of interest.
